# Talking Science

**DOI:** 10.1371/journal.pbio.0020210

**Published:** 2004-07-13

**Authors:** Ludmila Pollock

## Abstract

The Cold Spring Harbor Laboratory Oral History Collection presents interviews with over 80 leading scientists, drawn from the fields of molecular biology and genetics

*“…where people do come with a sense that there's going to be openness. That there's going to be a great comfort with people standing up and challenging a particular perspective. That young people are welcome. That old codgers are welcome as well. This is sort of the idyllic way that science is supposed to happen. And Cold Spring Harbor [Laboratory] provides that opportunity.”*

—Francis Collins, Cold Spring Harbor Laboratory Oral History Collection

Cold Spring Harbor Laboratory (CSHL) has played a significant role in the development of genetics, a role that began at the Laboratory in 1904, just a few years after the discovery of Mendel's work. In 1908, George Shull's development of hybrid corn (maize) shaped the future of modern agricultural genetics; 55 years ago, Barbara McClintock worked on maize cytogenetics and discovered “jumping genes”; and in 1977, Richard Roberts was a codiscoverer of “split genes.” Throughout the 1950s, the Phage Course at CSHL trained many of the leaders of the then new field of molecular genetics, and the Laboratory's courses continue to train young scientists.

CSHL Archives is a unique repository of rare books, manuscripts, photos, and scientific reprints that document the institution's significant contributions to molecular genetics research over the past 100 years. In 2000, the Archives received James Watson's personal collection of original materials, including manuscripts, correspondence, photographs, lab notes, lectures, memorabilia and reprints, for processing and preservation. This is an invaluable documentary resource for the history of molecular biology. While organizing over 60 years of Watson's correspondence, we realized that many of his correspondents have passed through CSHL as researchers, participants in the laboratory's leading scientific meetings, or instructors of our courses. We decided to talk to these scientists and record their commentaries, stories, and personal memories. These oral history interviews will enhance our collection by adding the voices of participants in significant scientific events.

The project began in 2000. In 2002, we received a grant from the Gladys Brook Foundation that enabled us to purchase state-of-the-art recording and editing equipment for carrying out this project, and created a web site exhibiting taped conversations. During the last three years, we have interviewed over 80 scientists from several generations working in different fields of molecular biology and genetics, and have developed a video collection at CSHL's Carnegie Library. (A customized DVD of this collection is available.)

The unique environment of the CSHL facilitated our task. Throughout the year, researchers from all over the world attend our scientific meetings to exchange information and ideas about their research, so most of the interviews took place at the Laboratory during these meetings. The scientists were always very pleasant and receptive to our requests for interviews. Other interviews were conducted in Cambridge and Boston, Massachusetts; La Jolla, California; Washington, DC; and abroad, in Oxford, England, and Sydney and Melbourne, Australia.

Interviewing scientists was very exciting. We often wanted to come back for an additional interview because one or two hours was not enough time to collect all the memories that they wanted to share with us. It is well known that Sydney Brenner is a superb storyteller. After spending a few hours listening to Sydney in La Jolla, California, I received a call confirming my meeting with Francis Crick, which was to take place in the next 30 minutes. Despite looking forward so much to this meeting, I was torn between my present interview, in which I was deeply involved, and my next, which I was gladly anticipating.

The CSHL Oral History Collection web site (library.cshl.edu) provides an opportunity to “meet and get to know” the leading minds of molecular biology and genetics. We hope that our site makes the history of science come to life. These interviews can be helpful to all categories of users: students, scientists, historians, writers, journalists, and others.

The site is searchable and allows visitors to cross-reference people and information, and links them to the world of narratives and anecdotes, as told in the voices of those interviewed. Selected interview clips are organized into five topics: CSHL, James D. Watson, prominent scientists, scientific experience, and genome research. Each topic is divided into subtopics; for example, “Genome Research” includes: involvement in genomics, mechanism of the Human Genome Project, challenges of the Human Genome Project, dangers of genomic research, the future of genomics, and others. “Woman in Science” (Figure 1) is one of the subtopics of “Scientific Experience.” Visitors also can choose a particular scientist and link to all the topics that he or she discussed. A brief biography of each participant has been included.

**Figure 1:**
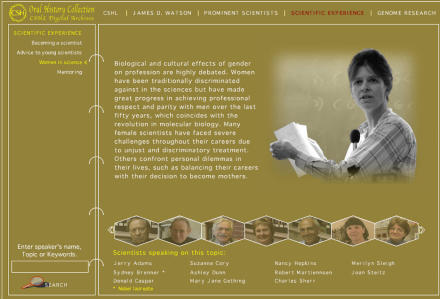
Women in Science One of the pages from the “Scientific Experience” topic of the CSHL Oral History Collection.

Our Web site adds a new dimension to the papers and books written by leading scientists—now you can listen to them discuss their work in their own voices! On this site you will meet a remarkable range of scientists, from friends and students of Barbara McClintock and Jim Watson, to the evolutionist Ernst Mayr, to the genome bioinformaticist James Kent. You'll hear Nancy Hopkins, Tom Maniatis, Matt Ridley, and Joan Steitz, to name just a few.

We remain committed to our objective of providing an unprecedented and exciting approach for scientists, historians, scholars, and students to gain a firsthand account of the history of molecular biology and the stories behind the people who contributed to it. We have many more interviews planned, so in the future you can expect to meet even more of the fascinating figures who illuminate the glory days of the world of molecular biology.

## Abbreviation

CSHL: Cold Spring Harbor Laboratory

